# Cleavage of proteoglycans, plasma proteins and the platelet-derived growth factor receptor in the hemorrhagic process induced by snake venom metalloproteinases

**DOI:** 10.1038/s41598-020-69396-y

**Published:** 2020-07-31

**Authors:** Amanda F. Asega, Milene C. Menezes, Dilza Trevisan-Silva, Daniela Cajado-Carvalho, Luciana Bertholim, Ana K. Oliveira, André Zelanis, Solange M. T. Serrano

**Affiliations:** 1 Laboratório de Toxinologia Aplicada, Center of Toxins, Immune-Response and Cell Signaling (CeTICS), Instituto Butantan, Av. Vital Brasil 1500, São Paulo, SP 05503-900 Brazil; 2 Departamento de Ciência e Tecnologia, Universidade Federal de São Paulo, São José dos Campos, SP Brazil; 3 Present Address: Laboratório Nacional de Biociências (LNBio), Centro Nacional de Pesquisa em Energia e Materiais (CNPEM), Campinas, São Paulo Brazil

**Keywords:** Biochemistry, Cell biology, Molecular biology

## Abstract

Envenoming by viperid snakes results in a complex pattern of tissue damage, including hemorrhage, which in severe cases may lead to permanent sequelae. Snake venom metalloproteinases (SVMPs) are main players in this pathogenesis, acting synergistically upon different mammalian proteomes. Hemorrhagic Factor 3 (HF3), a P-III class SVMP from *Bothrops jararaca*, induces severe local hemorrhage at pmol doses in a murine model. Our hypothesis is that in a complex scenario of tissue damage, HF3 triggers proteolytic cascades by acting on a partially known substrate repertoire. Here, we focused on the hypothesis that different proteoglycans, plasma proteins, and the platelet derived growth factor receptor (PDGFR) could be involved in the HF3-induced hemorrhagic process. In surface plasmon resonance assays, various proteoglycans were demonstrated to interact with HF3, and their incubation with HF3 showed degradation or limited proteolysis. Likewise, Western blot analysis showed in vivo degradation of biglycan, decorin, glypican, lumican and syndecan in the HF3-induced hemorrhagic process. Moreover, antithrombin III, complement components C3 and C4, factor II and plasminogen were cleaved in vitro by HF3. Notably, HF3 cleaved PDGFR (alpha and beta) and PDGF in vitro, while both receptor forms were detected as cleaved in vivo in the hemorrhagic process induced by HF3. These findings outline the multifactorial character of SVMP-induced tissue damage, including the transient activation of tissue proteinases, and underscore for the first time that endothelial glycocalyx proteoglycans and PDGFR are targets of SVMPs in the disruption of microvasculature integrity and generation of hemorrhage.

## Introduction

Envenoming resulting from snakebites is a neglected public health problem, involving death and disability, in many tropical and subtropical countries. Local or systemic hemorrhage and coagulopathy are primary manifestations of viperid envenoming, involving the synergistic effects of snake venom metalloproteinases (SVMPs) on plasma proteins, platelets and blood vessels. SVMPs are zinc-dependent enzymes classified in the M12B subfamily of metalloproteinases, in which the P-III class is distinguished by being comprised of proproteinase, proteinase, disintegrin-like, and cysteine-rich domains^1^. The proteinase domain of hemorrhagic SVMPs plays a role in the hydrolysis of specific capillary basement membranes, cell surface proteins, and extracellular matrix (ECM) proteins, promoting capillary rupture and content extravasation, and resulting in local or systemic hemorrhage^1–5^. However, various studies have shown that the disintegrin-like/cysteine-rich domains of SVMPs play a role in the engagement of the metalloproteinase with its substrates^6–8^. The mechanism proposed to explain the generation of hemorrhage by SVMPs involves the hydrolysis of specific proteins in the microvessel basement membrane causing its mechanical instability and culminating with disruption and extravasation^4,9^. A number of studies employing different molecular technologies have revealed some important substrates of SVMPs both in vitro and in vivo^2,5,10,11^, nevertheless, the key components of different cell membranes that are susceptible to limited proteolysis or degradation by SVMPs remain elusive.

The ECM is generally divided into the cell-adjacent basement membrane (BM) and the interstitial matrix. Two main classes of proteins are present in the ECM: proteoglycans, glycoproteins and fibrous proteins^12–15^. Capillary ECM provides structural support to blood vessels and regulates properties of endothelial cells and pericytes. The major components of the BM are collagens IV and XVIII, laminins, heparan sulfate proteoglycans and nidogen, whose organization and scaffolding provide proper capillary integrity and maintenance of vascular homeostasis^15^. Among the components of BM, proteoglycans are heavily glycosylated proteins containing at least one attached glycosaminoglycan chain^16^. In vascular endothelium tissue, permeability is controlled by the microvascular wall, comprised of the endothelial glycocalyx, the endothelium, BM, interstitial membrane and the cells surrounding the outer surface of the microvessel (pericytes and smooth muscle cells)^17–19^. In this context, the mesh-like structure of the glycocalyx, composed of a complex mixture of proteoglycans, glycoproteins, enzymes, heparan sulphate and hyaluronic acid, plays an important role in endothelial cell mechanotransduction of shear stress and is a key regulator of vascular permeability, cell adhesion, and inflammation^19,20^. Furthermore, degradation or shedding of endothelial glycocalyx components has been associated with bleeding and microvascular barrier dysregulation^21–23^.

Once snake venoms reach the blood circulation, plasma proteins are direct targets of their proteinases, including the coagulation cascade and complement components, proteinase inhibitors and kinin precursors^24–28^. In mammals, a number of SVMPs have been shown to clearly escape inhibition by plasma proteinase inhibitors, such as alpha-2-macroglobulin, and are capable of exerting their catalytic activities on a variety of macromolecular substrates^29–32^. In this context, SVMPs contribute to the coagulopathy characteristic of viperid envenomings, and a number of P-III SVMPs were shown to activate or degrade some components of the coagulant cascade, such as fibrinogen, factor II (prothrombin) and factor X, tissue factor and von Willebrand factor^27,33,34^.

Platelet-derived growth factor (PDGF), a potent mitogen synthesized and released by platelets, endothelial cells and macrophages, is a glycoprotein occurring in five isoforms (PDGF-AA, PDGF-AB, PDGF-BB, PDGF-CC and PDGF-DD), which participate in the regulation of cell growth and division^35–38^. The PDGF receptors (PDGFR-alpha and -beta) are cell surface class III tyrosine kinase proteins characterized by a 5-Ig-domain extracellular segment (designated D1–D5) and a split intracellular kinase domain^39^. Upon binding of ligand, the receptor subunits alpha and beta homodimerize or heterodimerize, undergo autophosphorylation, and activate downstream signal transduction involving SH-2-domain-containing molecules, with further internalization and degradation to quench the signal^40,41^. PDGFR-alpha signaling controls the development of various organs, whereas PDGFR-beta signaling plays a role in early hematopoiesis and blood vessel formation^42^. Knockout studies in mice revealed the importance of PDGFR-beta signaling in embryonic development, particularly in vasculogenesis and angiogenesis, as PDGFR-beta knockout mice died due to widespread hemorrhage and edema formation^43,44^. Similarly, it has been shown that the increase in PDGF signaling by a single point mutation in PDGFR-beta was sufficient to increase the early proliferative phase of injury response^45^.

HF3 is a potent hemorrhagic SVMP present in *Bothrops jararaca* venom. It shows a minimum hemorrhagic dose (MHD) of 15 ng (0.2 pmol) on the rabbit skin and 160 ng (2.3 pmol) on the mouse skin^46,47^. The mature form of HF3 is composed of 416 amino acid residues, including five putative *N*-glycosylation sites, with a calculated molecular mass of 46,317.2 Da implying that ~ 35% of its molecular mass as assessed by SDS-PAGE (~ 70 kDa) is accounted for as glycan chains^2,47,48^. The non-catalytic domains (disintegrin-like and cysteine-rich) of HF3 are involved in its functional activities displayed upon cells and tissues, such as the inhibition of collagen-induced platelet aggregation, the activation of macrophage phagocytosis mediated by integrin αMβ2, and the promotion of inflammation by increasing leukocyte rolling in the microcirculation^2,48–51^. Furthermore, HF3 was shown to degrade fibrinogen, fibronectin, vitronectin, von Willebrand factor, collagens IV and VI, laminin and Matrigel in vitro^2^, while the determination of its primary specificity using the Proteomic Identification of Cleavage Sites (PICS) approach showed that it prefers Leu residues at P1′ position^52,53^. The proteomic analysis of the hemorrhagic process generated by HF3 on the mouse skin revealed the hydrolysis of intracellular, extracellular, and plasma proteins, including some proteoglycans^11^. Moreover, the proteolytic activity of HF3 is not affected by plasma inhibitors, whereas α2-macroglobulin is cleaved by HF3^31^. Recently, the analysis of proteomic and peptidomic profiles of C2C12 myotubes treated with a sub-cytotoxic dose of HF3 revealed differential abundance of cell proteins involved in oxidative stress and inflammation, and proteolysis in the culture supernatant, indicating potential new substrates of HF3^54^. Furthermore, we showed that in the secretome of mouse embryonic fibroblasts, HF3 alters the N-terminome by promoting the cleavage of proteins of the ECM and of focal adhesions and the cysteine protease inhibitor cystatin-C^53^. The aim of this study was to gain new insights into the mechanisms of hemorrhage production by HF3 by expanding the analysis of the substrate repertoire of this SVMP.

## Results

Blood vessel damage resulting in extravasation contributes to local tissue damage and poor tissue regeneration observed upon viperid envenoming. This pathogenesis involves the disruption of hemostasis and hemorrhage, where metalloproteinases play key roles in different pathways. SVMPs may exert pro- or anti-coagulation effects by limited proteolysis or degradation of plasma components. Besides, SVMPs may cleave ECM proteins and microvasculature BM proteins leading to fluid extravasation into the surrounding tissue. The main objective of this study was to evaluate the role of proteins involved in the stability of ECM and integrity of glycocalyx (proteoglycans) and in hemostasis (blood coagulation proteins and PDGF/PDGFR) in the hemorrhage induced by HF3, thereby providing new insights into the mechanisms for the typical pathological effects observed upon envenoming.

### Interaction of HF3 with proteoglycans in vitro

**Figure 1:**
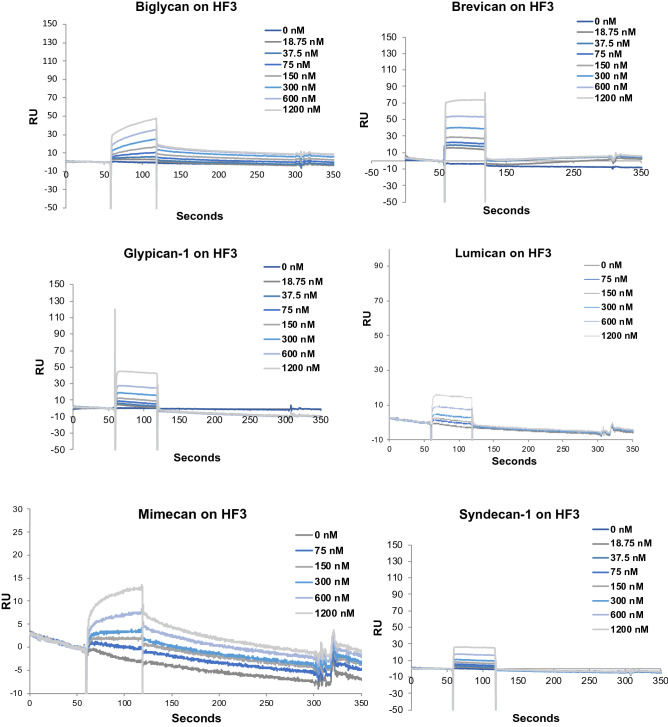
Interaction of HF3 with proteoglycans by surface plasmon resonance, using a Biacore T100 system. Proteoglycans were injected over immobilized HF3, at the indicated concentrations, as described in experimental procedures.

Proteoglycans are a complex group of proteins encoded by 43 genes^55^. Our aim with these studies was to investigate further the interaction of P-III class SVMPs with proteoglycans, including some components of the endothelial glycocalyx. Initially we tried to immobilize different proteoglycans to the BIAcore CM-5 sensorchip, however, without success (data not shown). To overcome this issue, HF3 was covalently immobilized on the BIAcore CM-5 sensorchip and assayed to assess its ability to bind to biglycan, brevican, glypican-1, lumican, mimecan and syndecan-1 using surface plasmon resonance (SPR). As observed in Fig. 1, these proteoglycans were capable of interacting with immobilized HF3 in a concentration-dependent fashion (75–1200 nM). On the other hand, although decorin was shown to be cleaved by HF3 in a previous study, using a decorin sample from a different source^11^, here it did not show interaction with HF3 at 1 µM and 5 µM (Supplementary Figure S1). In the case of aggrecan, we did succeed in covalently immobilizing it to a CM-5 sensorchip and HF3 was assayed for interaction to this proteoglycan at increasing concentrations (15–1000 nM), showing a concentration-dependent binding profile (Supplementary Figure S1).

### Proteolytic effect of HF3 on proteoglycans in vitro and in vivo

**Figure 2:**
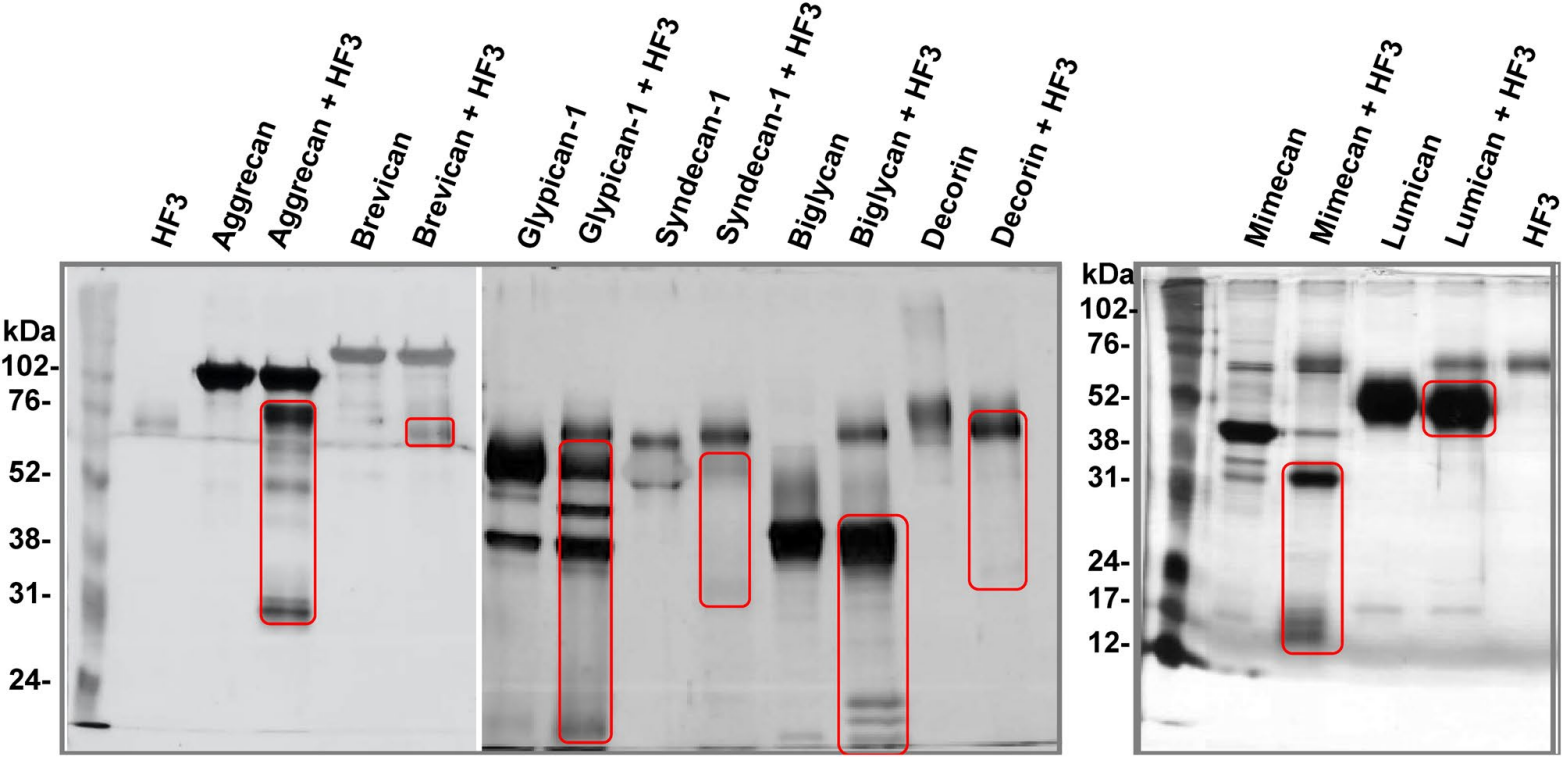
Activity of HF3 upon proteoglycans in vitro. Proteoglycans were incubated with HF3, as described in “Materials and methods”, and submitted SDS-PAGE. Proteins were stained with silver. Red rectangles indicate main degradation products.

Proteolysis of extracellular matrix and basement membrane components is considered to be a critical factor in the production of hemorrhage by certain SVMPs. Since HF3 showed interaction with various proteoglycans, we evaluated the ability of HF3 to catalyze their hydrolysis. For this purpose, HF3 was incubated with aggrecan, brevican, glypican-1, syndecan-1, biglycan, decorin, mimecan and lumican, as described in Experimental Procedures, and the reactions were further evaluated by SDS-PAGE (Fig. 2). Aggrecan, a chondroitin sulfate proteoglycan, is a major structural proteoglycan of the cartilage extracellular matrix, but is also expressed in the brain^55,56^. A recombinant aggrecan form composed of domains G1-IGD-G2 was seen as a band of ~ 120 kDa on the gel, and HF3 cleaved it generating fragments of 30 kDa, 50 kDa and 70 kDa. Brevican, another chondroitin sulfate proteoglycan, is one of the most important hyaluronan- and lectin-binding proteoglycans of the central nervous system^55,57^. It was nearly refractory to the proteolytic activity of HF3, however, a faint band of ~ 65 kDa was observed and may represent a hydrolysis product. Glypican-1 is a cell surface proteoglycan that contains heparan sulfate^55,58^ and was observed on the gel as a main ~ 60 kDa protein band and a secondary band of ~ 40 kDa. Upon incubation with HF3 it resulted in a single hydrolysis product of ~ 50 kDa while the band of ~ 40 kDa appeared with higher intensity indicating that it might also contain an additional hydrolysis product of similar molecular mass. Syndecan-1 is also a cell surface proteoglycan that contains both heparan and chondroitin sulfate and participates in the linkage of the cytoskeleton to the interstitial matrix^55,59^. The protein was observed on the gel at a molecular mass of ~ 60 kDa which nearly disappeared after incubation with HF3. Biglycan is a small leucine-rich repeat proteoglycan found in a variety of extracellular matrix tissues, which binds TGFβ^60^ and modulates its bioactivity^55,61^. Biglycan showed a molecular mass of ~ 45 kDa on the gel and was partially cleaved by HF3 resulting in products of ~ 24, 20, and 18 kDa. Decorin, a small leucine-rich proteoglycan that has the ability to bind and decorate fibrillar collagen in a periodic fashion^55,62^, migrated as a broad band of 80 kDa, which upon incubation with HF3 resulted in a product of ~ 75 kDa. Mimecan, or osteoglycin, is a small leucine-rich proteoglycan that induces ectopic bone formation in conjunction with transforming growth factor beta^55,63^. The 45 kDa band of mimecan was almost completely degraded to generate a main hydrolysis fragment of ~ 30 kDa and minor fragments of 13–15 kDa. Lumican is also a member of the small leucine-rich proteoglycan family^55,64^. The 55–60 kDa band of lumican showed a slight reduction of molecular mass to ~ 50 kDa indicating that HF3 may have promoted its limited proteolysis.

**Figure 3:**
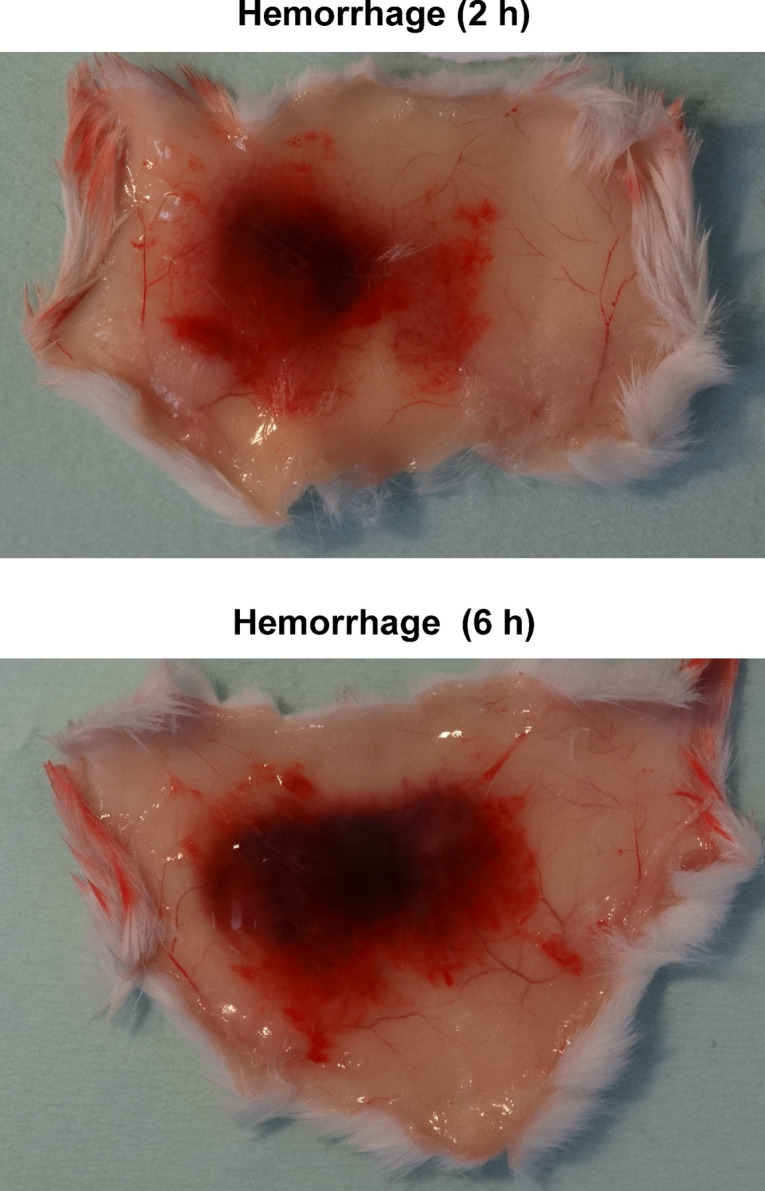
Representative images of hemorrhage generated in the mouse skin 2 h and 6 h after HF3 injection (1.0 μg in 50 mM HEPES, 1 mM CaCl_2_, pH 7.5), as described in “Materials and methods”.

We next examined whether endothelial glycocalyx-related proteoglycans could be degraded in vivo, in the hemorrhagic process generated after 2 h and 6 h of HF3 injection (1 µg; 14 pmol) in the mouse skin (Fig. 3).

**Figure 4:**
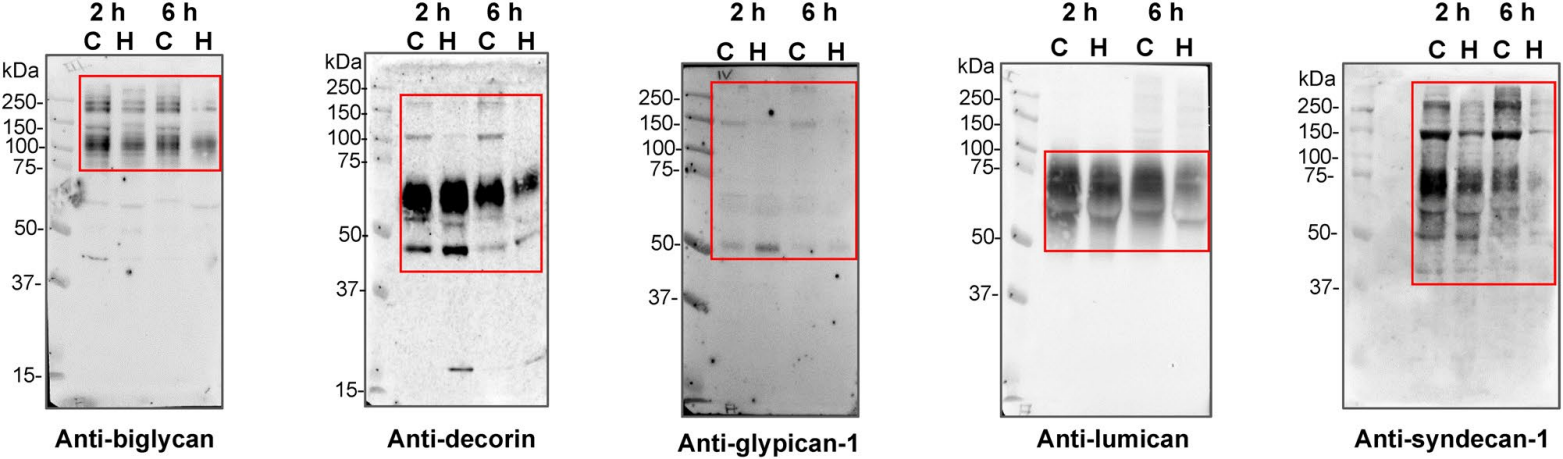
Degradation of glycocalyx-related proteoglycans in the mouse skin injected with HF3, as shown by Western blot analysis. Protein samples (30 μg) extracted from control skin (C) or skin injected with HF3 (H) after 2 h and 6 h, as described in “Materials and methods”, were immunostained with anti-biglycan, anti-decorin, anti-glypican-1, anti-lumican and anti-syndecan antibodies. Red rectangles indicate main protein bands recognized by the antibodies.

To this end, proteins extracted from mouse control and hemorrhagic skin were submitted to SDS-PAGE and immunostained with biglycan, decorin, glypican-1, lumican, and syndecan-1 antibodies. As seen in Fig. 4, in most cases, the degradation of these proteoglycans in vivo was more pronounced after 6 h of HF3 injection. Anti-biglycan antibody recognized protein bands of nearly 250 and 120 kDa, whose intensity clearly decreased in the hemorrhagic skin. In the control skin, anti-decorin antibody weakly immunostained two protein bands of ~ 170 and 120 kDa, while two strongly recognized bands showed 70 and 40 kDa. All bands recognized by anti-decorin either disappeared or diminished in intensity in the hemorrhagic skin. Immunodetection with an anti-glypican-1 antibody revealed protein bands of ~ 150 and 50 kDa in the control samples, which were not recognized in the hemorrhagic skin after 6 h of HF3 injection. In the case of lumican, a large smear of 60–75 kDa was recognized by the antibody, however with a core stained band of ~ 60 kDa, which decreased upon the 6 h hemorrhagic process and showed a slight molecular mass shift to ~ 58 kDa, as observed in Fig. 2. Anti-syndecan-1 recognized different bands (250, 150, 70–75 and 50–60 kDa), which nearly disappeared after 6 h of HF3 injection. Although aggrecan, brevican, and mimecan were cleaved in vitro by HF3, our results using antibodies to probe their abundance in the hemorrhagic skin were inconclusive to suggest a clear degradation of these proteoglycans in vivo (Supplementary Figure S2). Therefore, while it is not possible to make a direct comparison of the results on the cleavage of recombinant proteoglycans in vitro by HF3 and the analysis by Western blot using specific antibodies to assess the abundance of the same proteoglycans in the hemorrhagic skin, our results show a good agreement between the two approaches, and point out the hydrolysis of proteoglycans as a relevant mechanism for microvasculature destabilization and generation of hemorrhage.

### Gelatinolytic activity is generated in the mouse skin injected with HF3

**Figure 5:**
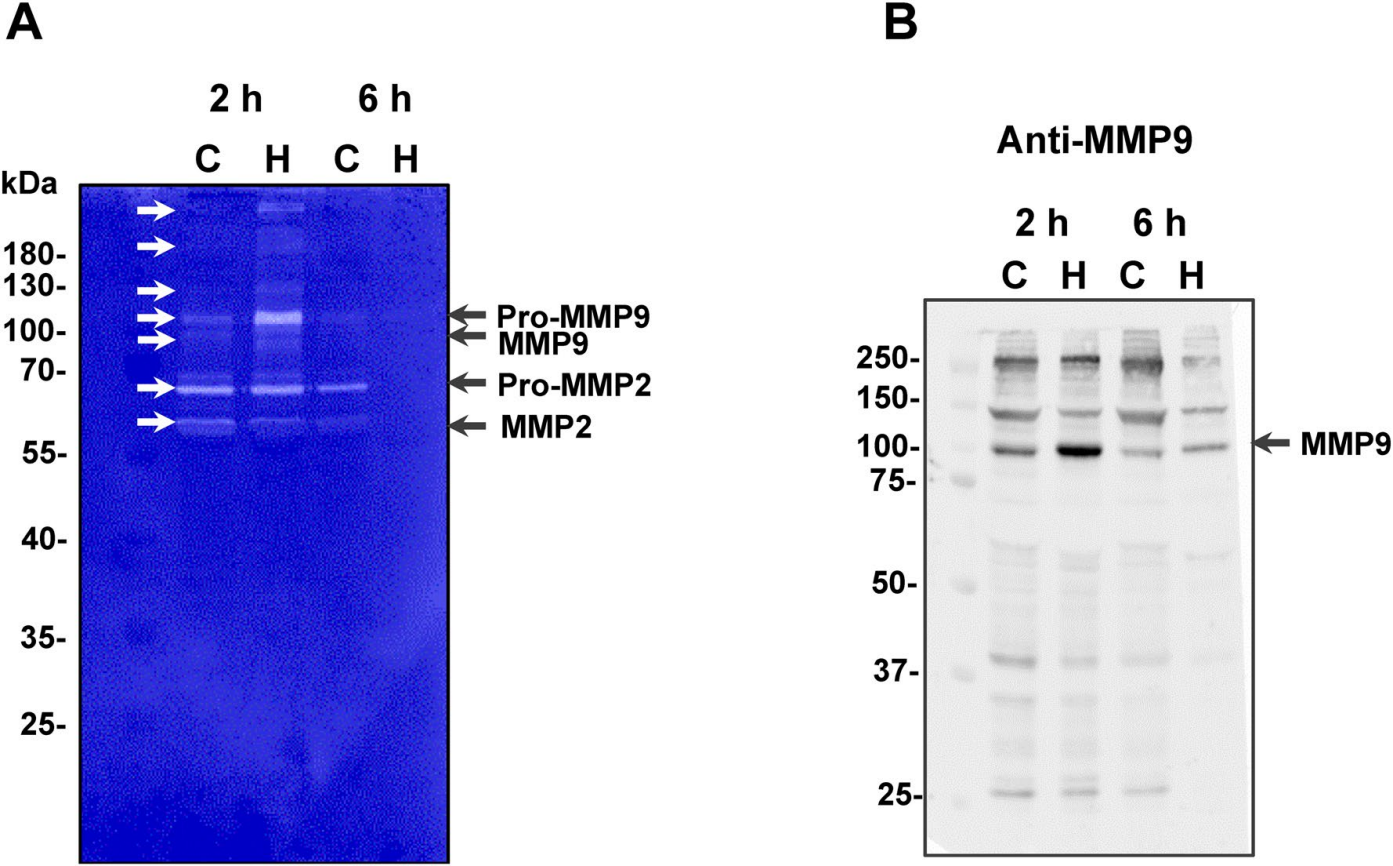
Activation of mouse skin proteinases. (**A**) Gelatin zymography of mouse skin proteins. Protein samples (30 μg) from control skin proteins (C) or hemorrhagic skin proteins (H), prepared under non-reducing conditions, were submitted to electrophoresis on 10% SDS–polyacrylamide copolymerized with gelatin. Gels were stained with Coomassie blue. Proteins with activity were identified as clear zones of lysis against a dark background. White arrows indicate bands of gelatinolytic activity. (**B**) Detection of the presence of MMP9 in the mouse skin, as shown by Western blot analysis. Protein samples (30 μg) from control skin proteins (C) or hemorrhagic skin proteins (H) were immunostained with anti-MMP9 antibodies. Numbers on the left indicate molecular mass marker mobility.

In a recent investigation on the effects of HF3 upon the secretome of mouse embryonic fibroblasts using terminal amine isotopic labeling of substrates (TAILS) analysis, we identified 190 unique peptide cleavages which suggested the participation of other proteinases activated upon incubation with HF3, including the matrix metalloproteinases (MMPs) 2 and 9^53^. Here, we assessed the possibility that these proteinases play a role in the proteolytic process affecting the ECM and endothelial glycocalyx proteoglycans in the hemorrhagic mouse skin (Fig. 3). Figure 5A shows the profile of gelatinolytic activity of skin proteins analyzed by zymography. Interestingly, the intensity of bands of ~ 68 kDa and ~ 58 kDa of gelatinolytic activity corresponding to, respectively, pro-MMP2 and active MMP2, were nearly similar in control and in the hemorrhagic skin after 2 h of HF3 injection, and disappeared after 6 h. However, a gelatinolytic band of ~ 105 kDa, corresponding to the zymogen of mouse MMP9, was detected as clearly more intense in the 2 h hemorrhagic process compared to the control, as well as and in its cleaved, activated form of 97 kDa. Interestingly, both bands were also absent in the skin within 6 h of hemorrhage, therefore, both MMP2 and MMP9 did not show any gelatinolytic activity in the hemorrhagic skin after 6 h of HF3 injection.

Immunostaining of the mouse skin proteins with an anti-MMP9 antibody revealed a band of ~ 100 kDa, corresponding to the recognition of MMP9, which was only more intense in the hemorrhagic skin 2 h after HF3 injection, in agreement with the gelatin zymography (Fig. 5B). This antibody also recognized protein bands of ~ 250 and 130 kDa which showed lower intensity in the 6 h hemorrhagic skin, however it is unclear whether these bands correspond to other molecular, possibly complexed, forms of MMP9.

### Proteolytic effect of HF3 on plasma proteins

**Figure 6:**
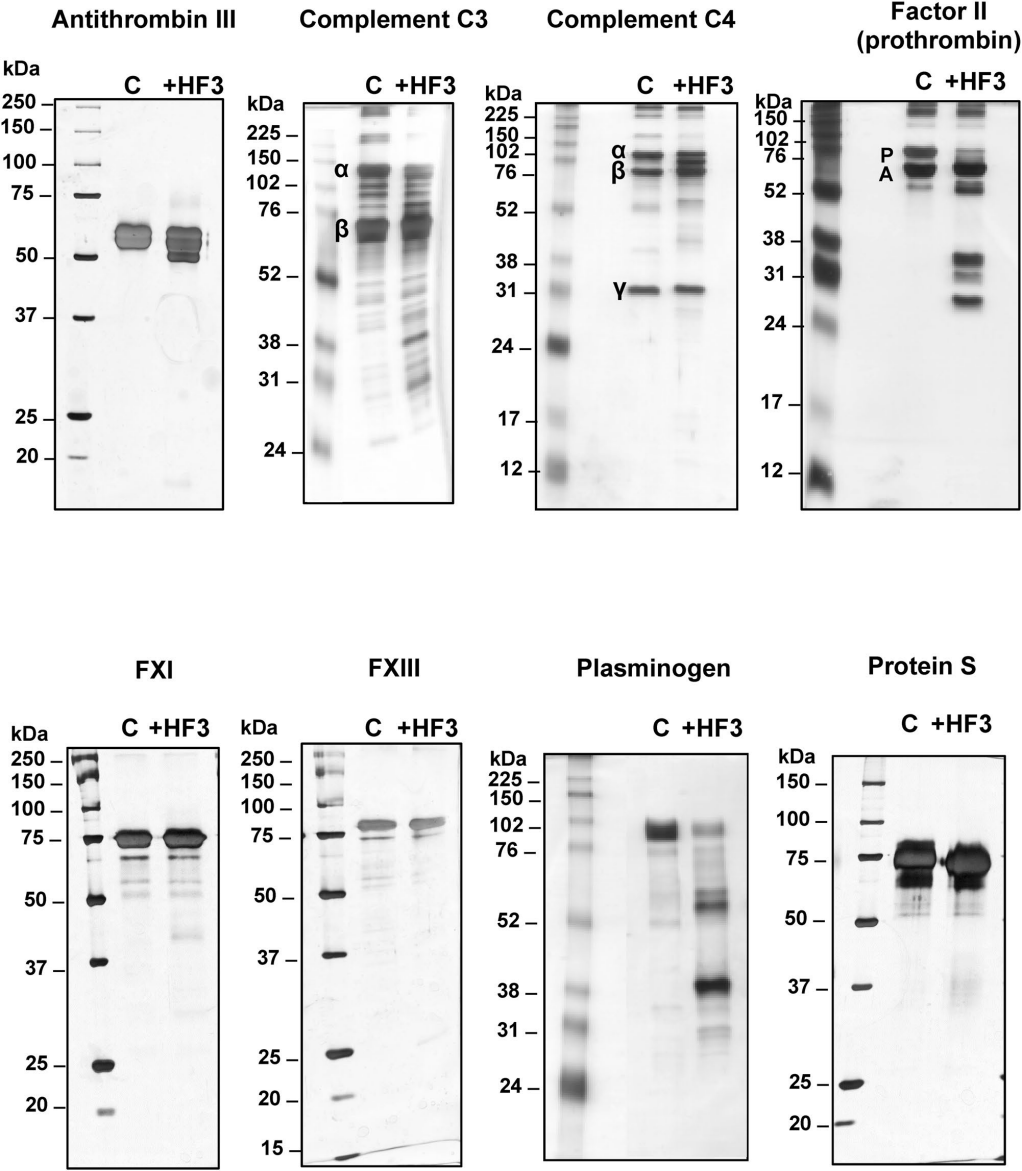
Activity of HF3 upon plasma proteins. Proteins were incubated with HF3 at a 1:10 (w/w) enzyme:substrate, as described in “Materials and methods”, and submitted SDS-PAGE. Greek letters indicate protein chains. P: prothrombin; A: albumin. Proteins were stained with silver.

The activity of SVMPs on plasma proteins is involved in the severe coagulopathy and local effects observed upon viperid envenoming. Among the plasma proteins, HF3 had previously shown to cleave alpha-2-macroglobulin, fibrinogen, fibronectin, vitronectin, and von Willebrand factor^2,31^. Here, we extended our analysis of plasma proteins to identify new substrates for HF3 by incubating antithrombin III, complement components C3 and C4, coagulation factors II (prothrombin), XI and XIII, plasminogen, and protein S with HF3 at a 1:10 (w/w) enzyme:substrate ratio and the reactions were further evaluated by SDS-PAGE (Fig. 6). Antithrombin III is a serine proteinase-inhibiting plasma protein (serpin) of 58 kDa that targets thrombin and other coagulation cascade components of the contact activation and tissue factor pathways^65^. HF3 promoted limited proteolysis of antithrombin III generating a product of ~ 50 kDa. Complement component C3 plays a central role in the activation of the complement system and its activation is required for both classical and alternative complement activation pathways^66^. Component C4 is cleaved by upstream proteinases of the complement cascade and converted into fragments C4a and C4b. C4b is a protein of 193 kDa which forms a complex with C2b, which in turn cleaves C3 into C3a and C3b as part of the events of the complement cascade. HF3 cleaved the complement C3α chain and generated various degradation products of ~ 25 to 50 kDa, while the C3β chain remained intact. As for component C4, after the incubation with HF3, although its C4α, C4β, and C4γ chains apparently remained intact, a main degradation product of ~ 85 kDa and two faint degradation products of ~ 40 kDa e 55 kDa were observed. Factor II (prothrombin) is a vitamin K-dependent coagulation component that upon activation is proteolytically cleaved to form thrombin, a serine proteinase that converts fibrinogen into fibrin^67^. The 72 kDa protein chain of prothrombin was almost completed degraded by HF3 to generate four stable products of ~ 28, 30, 35 and 50 kDa, of which the bands of 50 kDa and 30 kDa likely correspond to, respectively meizothrombin and thrombin, in a profile similar to that obtained by Morita and colleagues^68^ for the activation of prothrombin with the prothrombin activator from *Echis carinatus* venom. Factor XI (thromboplastin), which acts by cleaving factor IX in the intrinsic blood coagulation pathway, is a homodimer consisting of two identical subunits of 80 kDa held together by disulfide bonds^69^. Factor XIII, an enzyme with transglutaminase activity that crosslinks fibrin, is a tetramer of noncovalently associated pairs of subunits of 75 and 88 kDa^70^. Both factors XI and XIII were not cleaved upon incubation with HF3. Plasminogen is the inactive zymogen form of the serine proteinase plasmin, a fibrinolytic component of the coagulation cascade. After incubation with HF3, the 92 kDa band of plasminogen appeared with reduced intensity and two main products of 38 and 55 kDa were observed on the gel. The 38 kDa protein band might correspond to angiostatin, an internal proteolytic fragment of plasminogen that is an inhibitor of proliferation of endothelial cells^71^. Protein S, a single-chain, 69 kDa protein that plays a role in the anticoagulation pathway by acting as a cofactor to protein C in the inactivation of factors Va and VIIIa^72^ was not cleaved by HF3.

### Proteolytic effect of HF3 on PDGFR and PDGF in vitro and in vivo

**Figure 7:**
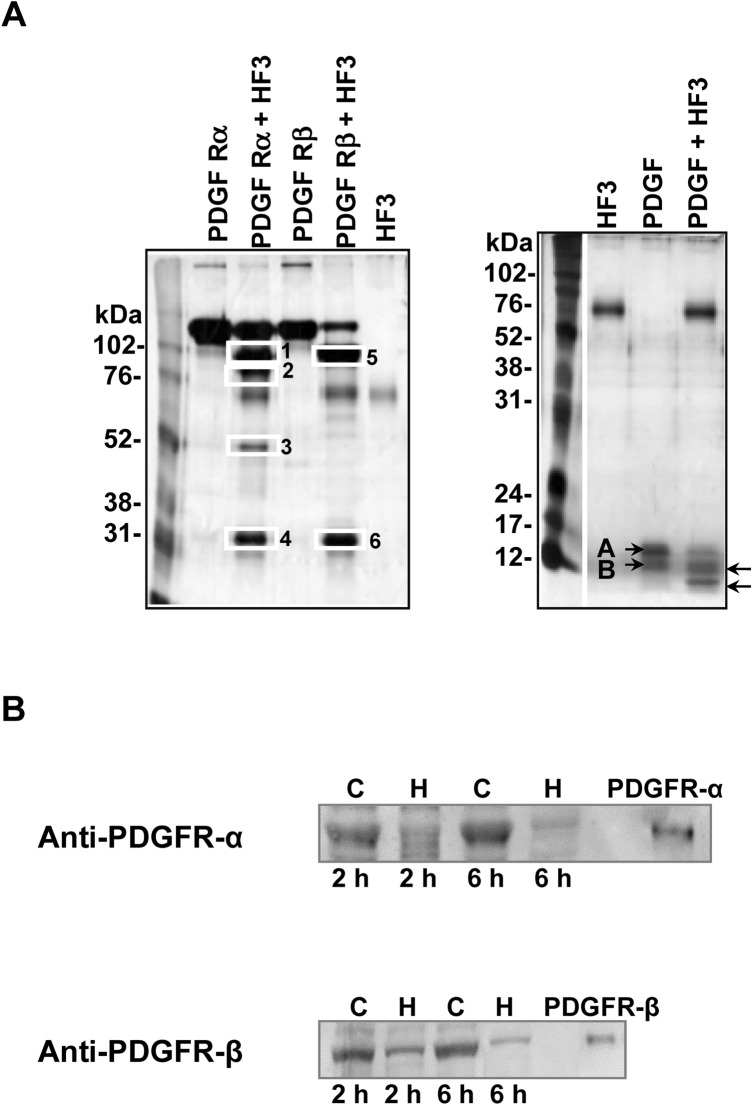
Activity of HF3 upon PDGFR and PDGF. (**A**) HF3 cleaves PDGFR (alpha and beta forms) and PDGF in vitro. PDGFR-α (recombinant mouse PDGFR-α-Fc chimera), PDGFR-β (recombinant mouse PDGFR-β-Fc chimera) and PDGF were incubated with HF3, as described in “Materials and methods”, and submitted SDS-PAGE. White rectangles indicate proteins bands identified by mass spectrometry. Letters A and B indicate the subunits of the disulfide­linked PDGF heterodimer. Arrows indicate cleavage products generated by HF3. Proteins were stained with silver. (**B**) Degradation of PDGFR-α and PDGFR-β in the mouse skin injected with HF3, as shown by Western blot analysis. Protein samples (30 μg) from control skin (C) or skin injected with HF3 (H), and PDGFR-α (recombinant mouse PDGFR-α-Fc chimera) and PDGFR-β (recombinant mouse PDGFR-β-Fc chimera) (10 ng) were immunostained with anti-PDGFR-α and anti-PDGFR-β antibodies. Full-length images of Western blots and SDS-PAGE profiles of mouse proteins from control and hemorrhagic skins in comparison with PDGFRα and PDGFRβ are shown in Supplementary Figure S3.

The PDGF/PDGFR axis has been shown to participate in signaling events that control a spectrum of cellular responses in both physiological and pathological scenarios^41^. To examine whether PDGF/PDGFR could be involved in the hemorrhagic process induced by HF3, we incubated PDGFR-α chimera, a recombinant mouse protein composed of mouse PDGFRα (Leu25­Glu524) linked via the peptide IEGRMD to the human IgG1 (Pro100­Lys330), and PDGFR-β chimera, a recombinant mouse protein composed of mouse PDGFRβ (Leu32­Glu530) linked via the peptide IEGRMD to the human IgG1 (Pro100­Lys330), with HF3 at a 1:10 (w/w) enzyme:substrate ratio. As seen in Fig. 7A, PDGFR-α chimera and PDGFRβ-chimera showed specific, limited proteolysis by HF3. Cleavage products from both proteins following incubation with HF3 when subjected to mass spectrometric analysis indicated that the sites of cleavage in the PDGFR-chimeras were localized to regions within the PDGFRs, while products originated from the IgG1 region were not detected (Supplementary Tables S1 and S2). HF3 was also incubated with human PDGF, a protein consisting predominantly of PDGF-AB heterodimers, at a 1:10 enzyme:substrate ratio. The protein migrated on the gel as close bands of ~ 14 and 12 kDa and was cleaved by HF3 generating products of ~ 10 and 8 kDa (Fig. 7A). We next examined whether the PDGFRs could be degraded in vivo, in the hemorrhagic process generated by HF3 in the mouse skin (Fig. 3). As seen in Fig. 7B, PDGFRα and PDGFRβ were degraded in the mouse skin, as shown by Western blot analysis of skin proteins after 2 h and 6 h of injection with HF3, with more pronounced effect observed after 6 h.

## Discussion

Over the past several years there has been mounting evidence showing the correlation of snake venom-induced pathologies with the activity of metalloproteinases and pointing out to these venom components as potent factors for the perturbation of the prey’s or human victim circulatory system, with the endothelium and the blood components being directly affected by the venom. In this context, the hemorrhagic process generated by SVMPs is a complex phenomenon resulting in capillary disruption and blood extravasation. Here, we focused on the hypothesis that various proteoglycans, especially those of the endothelium glycocalyx, plasma proteins, and PDGFR/PDGF could be involved in the hemorrhagic process induced by HF3 and hence carried out both in vivo and in vitro assays.

Basement membrane proteoglycans are found at the periphery of cells and those associated with vascular endothelial cells play important roles in endothelial functions, such as in the inhibition of intravascular coagulation and extravasation of plasma proteins and blood cells^73^. The endothelium is lined with a glycocalyx layer that functions as a barrier between circulating blood and the vessel wall. SVMPs have been shown to be active upon a variety of substrates, including plasma proteins, extracellular matrix components located in the basement membrane of the microvasculature, endothelial cells and components involved on platelet-aggregation. In the scenario of local hemorrhage induced by SVMPs there appears to be a clearing of regions of capillary basement membrane with a subsequent dissolution of the endothelial lining, ultimately allowing escape of capillary contents into the stroma^74^. In this context, the ability of SVMPs to degrade proteoglycans may be a relevant mechanism to induce hemorrhage. Tortorella and colleagues^75^ showed for the first time that a P-I class SVMP (atrolysin C, from *Crotalus atrox* venom) was able to cleave aggrecan in vitro. Moreover, Escalante and colleagues^76^ have shown that perlecan was present in the exudate collected from muscle tissue injected with BaPI, from *B. asper* venom. Using proteomic analysis, we have shown that in the hemorrhage generated by HF3 in the mouse skin there is degradation of decorin, lumican and mimecan^11^. Here, using SPR we show that HF3 dose-dependently interacted with proteoglycans aggrecan, brevican, glypican-1, syndecan-1, biglycan, mimecan and lumican, but not with decorin. Nevertheless, decorin, and all the other proteoglycans were susceptible to degradation or apparent limited proteolysis by HF3. Furthermore, immunostaining with specific antibodies showed the degradation of proteoglycans composing the endothelial glycocalyx (biglycan, decorin, glypican-1, lumican, and syndecan-1) in the hemorrhagic process generated by HF3 in the mouse skin.

Although at different levels, all proteoglycans tested in this study are detected in the skin either as mRNA or protein (https://www.proteinatlas.org/)^77^. Therefore, their direct in vitro degradation by HF3, or in vivo, in the hemorrhagic process generated by HF3, suggests a critical role of the destabilization of the mouse skin integrity promoted by HF3, as noticed by the extreme friability of the hemorrhagic skin tissue observed upon cryostat sectioning for histological analysis (not shown). Notably, the results of this study underscore the role of degradation of biglycan (extracellular matrix), decorin (pericellular matrix), glypican (cell surface), lumican (extracellular matrix) and syndecan-1 (cell surface) in the local hemorrhagic process by SVMPs. Biglycan degradation in the hemorrhagic process would have twofold implications, as this proteoglycan (1) participates in different proinflammatory signaling pathways in conjunction with multi-receptor crosstalk in macrophages^78^, and (2) can be released from the ECM by proteolytic enzymes of the MMP family, or by granzyme B^79–82^ then acting as a Damage-Associated Molecular Pattern (DAMP) molecule^78,83,84^. Decorin plays a role in the regulation of collagen fibrillogenesis and functions as a reservoir of transforming growth factor beta 1 (TGF-beta) in the extracellular matrix^85–87^ implying that in the hemorrhagic process induced by HF3 decorin cleavage might induce tissue reactions mediated by TGF-beta released in the connective tissue. Glypicans are bound to the outer surface of the cell membrane by a glycosyl-phosphatidylinositol anchor, while their heparan sulfate chains bind growth factors and participate in cell signaling, proliferation and matrix production^88^. For instance, glypican-1, by means of its heparan sulfate chains, regulates the stability of the interaction of fibroblast growth factor 2 (FGF2) and its receptor, protecting FGF2 from degradation^89^. The shedding of glypican-1 by ADAM 17 was recently reported^90^, and its degradation in the hemorrhagic process induced by HF3 suggests that it is susceptible to proteolysis with implications to growth factors functions in physiological and pathological conditions. Lumican has been shown to be cleaved by MT-MMP1^91^ and MMP13^79^. The interaction of lumican with collagen fibers at sites prone to their cleavage by collagenases was shown to sterically retards or protects against proteolysis^92^, suggesting that in the hemorrhagic process induced by HF3, collagens would be more susceptible to degradation. Cell-surface syndecans links the cytoskeleton to the interstitial matrix by means of heparan sulfate chains and is expressed on leukocytes and endothelial cells. Syndecans regulate many cellular processes, such as adhesion, proliferation, and migration, and are susceptible to shedding by proteinases, resulting in the release of ectodomains that can act as competitive inhibitors of their cell surface-linked counterparts^93–95^ and modulate the onset of different pathological processes^96–98^. Considering the role of syndecan-1 in the endothelial glycocalyx, its degradation in the hemorrhagic process generated by HF3 in the mouse skin suggests the impairment of its functions with consequences related to glycocalyx permeability and mechanotransduction of fluid shear stress, as well as to leukocyte access to endothelial tissue.

While we show that HF3 can directly interact with and cleave proteoglycans in vitro, the possibility that tissue proteinases may also contribute to their degradation in the HF3-induced hemorrhage cannot be ruled out. Indeed, proteoglycans are susceptible to shed or degradation by different metalloproteinases (MMP, MT-MMP, ADAM and ADAMTS)^99–103^ and therefore the increased MMP9-like gelatinolytic activity detected among proteins extracted from the hemorrhagic tissue 2 h after HF3 injection points out to the dysregulation of homeostasis and proteolytic balance triggered by protein degradation products, cytokines and other inflammation mediators such as DAMP molecules, with modulation of the tissue proteolytic signature. Because of their potential for tissue damage, there are different ways to regulate MMPs activity, including gene expression, zymogen activation, and enzyme inactivation by specific inhibitors. Interestingly, the increased gelatinolytic activity in the 2 h hemorrhagic process was a transient phenomenon, which took a turn and evolved to an unexpected absence of activity within 6 h of hemorrhagic process, indicating a clear tissue response to proteolytic damage.

Regarding the hemorrhagic process induced by SVMPs, it has been suggested that the degradation of capillary basement membrane results in the weakening of the mechanical stability of the capillary wall, causing its distention due to the action of hemodynamic biophysical forces operating in the circulation and resulting in the capillary wall disruption and extravasation of blood^5,9^. As the glycocalyx is suggested to play a role in the regulation of vascular permeability, in the prevention of the adhesion of blood cells to the vessel wall, and in the transmission of shear stress, our findings suggest that in vivo the activity of SVMPs, not only on basement membrane components but also on the endothelial glycocalyx would result in disruption of microvessel integrity and dynamics, and contribute to local inflammation and hemorrhage. Besides, it is also tempting to speculate that the degradation products originated from the activity of SVMPs in the endothelial glycocalyx microenvironment could interfere with the microvascular barrier regulation and have a direct effect upon the permeability of endothelial cells.

The mechanisms involved in the hemorrhagic process generated by HF3, particularly the role of the cleavage of plasma proteins in the context of the hemorrhage, remain not fully understood. HF3 had been previously shown to directly cleave alpha-2-macroglobulin, fibrinogen, fibronectin, vitronectin, and von Willebrand factor in vitro^2,31^. Moreover, we showed that alpha-1-antitrypsin, apolipoprotein AII, fibrinogen and fibronectin are degraded in the hemorrhagic process induced by HF3 in the mouse skin^11^. Here we show that HF3 is also able to cleavage antithrombin III, components C3 and C4 of the complement system, factor II and plasminogen, but not factors XI and XIII and protein S in vitro. Among these substrates, there are proteins of the coagulation cascade and of the complement system, as well as plasma proteinase inhibitors, indicating that HF3 escapes inhibition and may act in an unregulated fashion causing the imbalance of hemostasis. Cleavage of fibrinogen, factor II and plasminogen by HF3 indicate a clear interference of this SVMP in three strategic points of the coagulation cascade, i.e., the generation of fibrin, thrombin and plasmin. Pidde-Queiroz et al.^104^ showed the cleavage of complement components C3 and C4 by a P-I class SVMP from *B. pirajai* venom. The cleavage of these proteins in vitro by HF3 suggests the ability of HF3 to impair the proper immune response provided by activation the classical and alternative pathways of the complement cascade. Antithrombin is synthesized by endothelial cells and is found within the glycocalyx, where it exerts specific anti-inflammatory effects^105,106^. Furthermore, antithrombin III was shown to play a role in the preservation of the endothelial glycocalyx from shedding upon ischaemia/reperfusion^106,107^. A previous report had shown the proteolytic inactivation of antithrombin III by a P-I class SVMP from *Crotalus adamanteus* (adamalysin) in the presence or absence of heparin^108^, generating a stable hydrolysis product similar to that observed with HF3. Our finding on the cleavage of antithrombin III by HF3 adds a novel mechanism by which SVMPs interfere with endothelial glycocalyx integrity contributing to hemorrhage and inflammation.

To the best of our knowledge, a role for the PDGF/PDGFR axis has not been assessed in the hemorrhagic process induced by SVMPs. Among the physiological processes in which the PDGF/PDGFR family participates, the migration and proliferation of pericytes contribute to the formation and proper function of blood vessels^109^. Likewise, a link between the activity and inhibition of PDGFR has been shown in different endothelial pathology scenarios. For instance, the PDGF signaling through PDGFRα and PDGFRβ induces angiogenesis by up-regulating vascular endothelial growth factor production and modulating the proliferation and recruitment of perivascular cells^110^. Furthermore, it has been shown that the disruption of PDGF-B/PDGFRβ paracrine signaling affects perivascular cells and endothelial cells proliferate irregularly, leading to improper vessel formation and hemorrhage^111^. And concerning the crucial role of PDGFRβ–mediated interactions in recruitment of mural cells by newly formed vessels, genetic-induced disruption of PDGF-B or PDGFRβ leads to the development of microvascular leakage, lethal hemorrhage, and edema in late embryogenesis in mice^112,113^. Recently, using a rat model, Hall et al.^114^ showed that the use of specific PDGFRβ inhibitors resulted in pericyte depletion and hemorrhage into the corpus luteum of the ovary. In line with these findings, here we show that HF3 is able to cleave PDGFR (alpha and beta forms) and PDGF in vitro. Interestingly, immunostaining of proteins extracted from the mouse hemorrhagic skin also showed the degradation of PDGFR (alpha and beta forms), suggesting that the concerted function of the PDGF-mediated signaling upon PDGFR may be impaired in the hemorrhagic process induced by HF3.

## Conclusions

**Figure 8:**
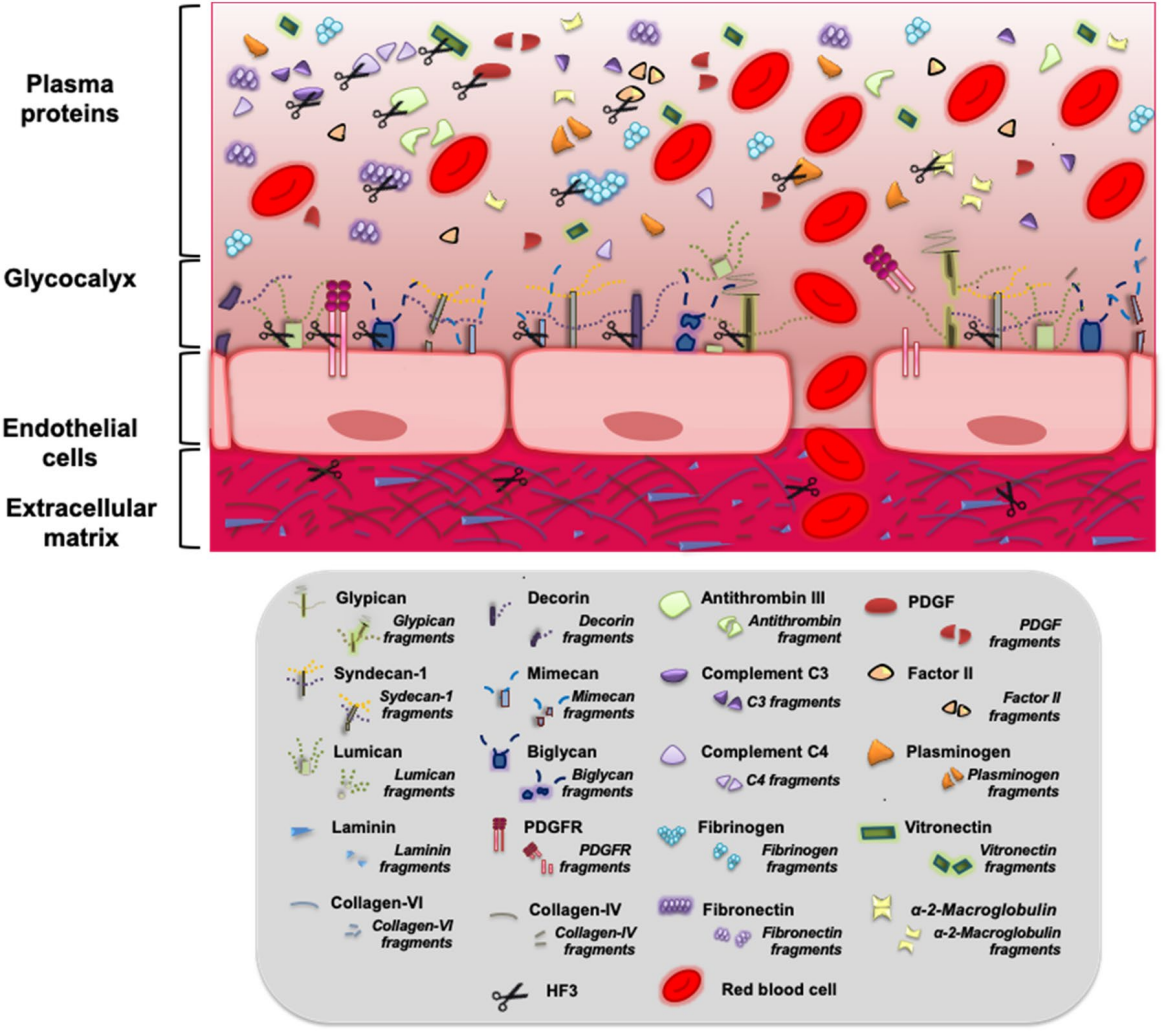
Schematic illustration of different proteins and proteoglycans that are cleaved in vitro by HF3. Components of the ECM, of the endothelial cell membrane, of the endothelial glycocalyx, and of plasma are potential targets of HF3, as reported in this and in former studies, and cited throughout this article. Some of these proteins and proteoglycans have also been shown to be cleaved in vivo, either due to the direct activity of HF3 or by tissue proteinases activated in the inflammatory/hemorrhagic scenario generated by HF3 in the mouse skin, culminating in the disturbance of hemostasis, and in the destabilization of the microvasculature via disruption of glycocalyx integrity, of cell–cell and cell–ECM interactions.

This investigation serves to underscores new targets of an extremely potent snake venom metalloproteinase that induces hemorrhage in the mouse and rabbit skins at pmolar, hormonal-like doses. Components of the endothelial glycocalyx, plasma proteins and PDGF/PDGFR are shown for the first time to be potentially involved in the generation of hemorrhage. Taken together, our results suggest that the effects of HF3 in the mouse skin and plasma are rather complex and involve the degradation or limited proteolysis of a range of substrates that might be directly hydrolyzed by HF3 or by tissue proteinases activated in the inflammatory/hemorrhagic scenario generated in vivo by HF3. In consonance with the two-step model proposed by Escalante and colleagues^4^, these events generate the triggering of tissue and plasma pathological responses, derived from the degradation of proteins and from their degradation products, that culminate in the disturbance of hemostasis, and in the destabilization of the microvasculature via disruption of glycocalyx integrity, of cell–cell and cell–ECM interactions (Fig. 8).

## Materials and methods

### HF3

HF3^48^ (Uniprot entry Q98UF9) was purified as described previously^47^ from *B. jararaca* venom provided by the Laboratory of Herpetology of Butantan Institute (São Paulo, Brazil). Briefly, the venom (900 mg) was dissolved in 90 mL of 0.15 M NaCl, centrifuged (4 °C, 10,000 g, 20 min), and submitted to saturation with ammonium sulfate. The protein fraction obtained between 30 and 60% of ammonium sulfate was dissolved in 20 mL water, dialyzed against 0.02 M Tris–HCl buffer containing 1 mM CaCl2 and 0.02% sodium azide (pH 7.5), at 4 °C, and chromatographed on a DEAE-cellulose DE-52 column previously equilibrated with the dialysis buffer, at 4 °C. The bound fraction, eluted with 0.15 M–0.20 M NaCl, contained HF3 and was concentrated by lyophilization, and resuspended with 0.25 M ammonium bicarbonate containing 1 mM CaCl2 and chromatographed in aliquots of 0.25 mL on a Superose 12 h 10/30 column (GE Healthcare), at 0.1 mL/min, using an Äkta Purifier system (GE Healthcare). HF3 was obtained in different peaks of the Superose 12 chromatography and identified by trypsin digestion and mass spectrometric (LC–MS/MS) analysis, as described before^47^.

### Proteolytic effect of HF3 on proteoglycans in vitro

Aggrecan (domains G1-IGD-G2), brevican, glypican-1, syndecan-1, byglican (R&D Systems) and decorin (Sigma-Aldrich) (2 µg) were incubated with 100 ng HF3 in 50 mM Tris–HCl, pH 8.0, containing 1 mM CaCl2 [1:20 (w/w) enzyme:substrate ratio; final volume 20 µL], for 3 h at 37 °C. Mimecan and lumican (R&D Systems) (2 µg) were incubated with 200 ng HF3 in 50 mM Tris–HCl, pH 8.0, containing 1 mM CaCl_2_ [1:10 (w/w) enzyme:substrate ratio; final volume 20 µL], for 3 h at 37 °C. A sample of each protein was incubated without HF3 under identical conditions. Reactions were interrupted by adding Laemmli sample buffer^115^ and submitted to SDS-PAGE under reducing conditions using different polyacrylamide concentrations: 10% (aggrecan, brevican, glypican-1, syndecan-1, byglican and decorin) and 12% (mimecan and lumican).

### Proteolytic effect of HF3 on plasma proteins in vitro

Antithrombin III (Abcam), complement components C3 and C4 (Complement Technology), coagulation factors II (prothrombin; Hyphen Biomed), XI and XIII (Abcam), plasminogen (Calbiochem), and protein S (Abcam) (2 µg) were incubated with HF3 (200 ng) in 50 mM Tris–HCl, pH 8.0, containing 1 mM CaCl2 [1:10 (w/w) enzyme:substrate ratio; final volume 20 µL], for 2 h at 37 °C. Reactions were interrupted by adding Laemmli sample buffer^115^ and submitted to SDS-PAGE under reducing conditions using different polyacrylamide concentrations (C3 and plasminogen, 10%; antithrombin III, C4, factors II, XI and XIII, and protein S, 12%).

### Proteolytic effect of HF3 on PDGFR and PDGF in vitro

PDGFR-α [recombinant mouse protein composed of mouse PDGFRα (Leu25­Glu524) linked via the peptide IEGRMD to the human IgG1 (Pro100­Lys330)], PDGFR-β [recombinant mouse protein composed of mouse PDGFRβ (Leu32­Glu530) linked via the peptide IEGRMD to the human IgG1 (Pro100­Lys330)] (2 µg), and PDGF (200 ng) (R&D Systems) were incubated with HF3 in 50 mM Tris–HCl, pH 8.0, containing 1 mM CaCl2 [1:10 (w/w) enzyme:substrate ratio; final volume 20 µL], for 3 h at 37 °C. Reactions were interrupted by adding Laemmli sample buffer^115^ and submitted to SDS-PAGE under reducing conditions using different polyacrylamide concentrations: 9% (PDGFR) and 12% (PDGF).

### Identification of PDGFR degradation products by mass spectrometry

Protein bands were excised, and in-gel trypsin digestion was performed according to Hanna and colleagues^116^. An aliquot (4.5 µL) of the resulting peptide mixture was desalinized using a C-18 column (180 μm × 20 mm) and separated in a C18 column (8 cm × 75 μm) (Waters) by RP-HPLC (Eksigent) coupled with a LTQ-XL mass spectrometer (Thermo Scientific) at a flow rate of 500 nL/min. The gradient was 2–80% acetonitrile in 0.1% formic acid over 30 min. The instrument was operated in the top ten mode, in which one MS spectrum is acquired followed by MS/MS of the top ten most-intense peaks detected, using 2.5 kV and 200 °C as source voltage and temperature, respectively. Full dynamic exclusion was used to enhance dynamic range—one spectrum before exclusion for 120 s. The resulting fragment spectra were searched using Mascot (version 2.2) search engine (Matrix Science) against nrNCBI Databank (www.ncbi.nlm.nih.gov) restricted to “Mus” taxonomy with a parent tolerance of 1.5 Da and fragment tolerance of 1.0 Da. Iodoacetamide derivative of cysteine and oxidation of methionine were specified in Mascot as fixed and variable modifications, respectively. Mascot identifications required ion scores greater than the associated identity scores and 20, 30, 40 and 40 for singly, doubly, triply, and quadruply charged peptides, respectively.

### Analysis of the interaction of HF3 with proteoglycans in vitro

Protein–protein interactions were assessed by surface plasmon resonance with a BIAcoreT100 system (GE Healthcare), as previously described^2^. HF3 and aggrecan were covalently immobilized on the BIAcore CM-5 sensorchip (carboxylated dextran matrix; GE Healthcare) according to the manufacturer’s instructions. Briefly, the CM-5 chip was activated with a 1:1 mixture of 0.4 M 1-ethyl-3-(3-dimethylaminopropyl) carbodiimide and 0.1 M N-hydroxysuccinimide for 7 min. HF3 (1 µM) and aggrecan (0.2 µM) in 10 mM sodium acetate, pH 4.0, were injected over the activated CM-5 chip at 25 °C. Remaining active groups on the matrix were blocked with 1 M ethanolamine/HCl, pH 8.5. Immobilization of HF3 and aggrecan on CM-5 sensorchip resulted in average surface concentrations of 2.45 ng/mm^2^ and 1.7 ng/mm^2^, respectively. Samples of proteoglycans biglycan, syndecan-1, glypican and brevican (0, 18.75, 37.5, 75, 150, 300, 600 and 1200 nM), mimecan and lumican (0, 75, 150, 300, 600 and 1200 nM) in HBS-EP buffer (10 mM HEPES pH 7.4, containing 150 mM NaCl, 3 mM EDTA and 0.005% surfactant P-20), were injected over immobilized HF3 at a flow rate of 30 µL/min. HF3 (0; 15,62; 31,25; 62,5; 125; 250; 500 and 1000 nM) in HBS-EP buffer was injected over immobilized aggrecan at a flow rate of 30 µL/min. Results were analyzed using BIAevaluation software version 1.1.1.

### Animals

Male Swiss mice were housed in temperature-controlled rooms and received water and food ad libitum. These studies were approved by the Experimental Animals Committee of Butantan Institute, São Paulo, Brazil; (Protocol 688-09), in accordance with guidelines of the National law for Laboratory Animal Experimentation Control (Law No. 11.794, October 8, 2008).

### Injection of HF3 in the mouse skin

Generation of hemorrhage in the mouse skin was carried out as previously described^2^. Briefly, male Swiss mice (n = 3) weighing 18–22 g were injected intradermally on the dorsal region with 100 μL of a control solution (50 mM HEPES, 1 mM CaCl_2_, pH 7.5) or with 100 μL of a solution containing 1.0 μg of HF3 in 50 mM HEPES, 1 mM CaCl_2_, pH 7.5. After 2 h and 6 h, animals were euthanized, and the dorsal skin was sectioned and homogenized with a tissue homogenizer (Polytron PT3100; Kinematica) using lysis buffer (50 mM HEPES, 200 mM NaCl, 2% CHAPS, pH 7.5) containing the HALT protease inhibitor cocktail (Thermo Fisher) and 5 mM EDTA (final concentration), with 18,000 rpm cycles, for 30 s, in ice bath. Homogenates were clarified by centrifugation at 14,000 × *g*, for 10 min, at 4 °C, to remove debris and the clear supernatant was carefully removed for analysis.

### Gelatin zymography of mouse skin proteins

Protein samples (30 μg) from mouse control skin or skin injected with HF3, prepared under nonreducing conditions, as described before^117^, were submitted to electrophoresis on 10% SDS-PAGE copolymerized with gelatin (1 mg/mL; Sigma-Aldrich). Gels were stained with Coomassie blue and destained. Gelatin digestion was identified as clear zones of lysis against a blue background.

### Western blot analysis of proteoglycans, MMP9 and PDGFRs in the mouse skin

Skin proteins from control and HF3-treated animals (30 μg), PDGFR-α (recombinant mouse PDGFR-α-Fc chimera), and PDGFR-β (recombinant mouse PDGFR-β-Fc chimera) (10 ng) were submitted to SDS-PAGE (10% SDS–polyacrylamide gel) under reducing conditions and transferred onto nitrocellulose membranes (GE Healthcare)^118^. Membranes were blocked for 3 h in PBS-T buffer (150 mM sodium chloride, 20 mM sodium phosphate, pH 7.1, and 0.1% Tween 20) containing 5% nonfat milk and were then incubated for 16 h, according to manufacturer’s instructions, with antibodies recognizing mouse lumican (AF2745; R&D Systems), mimecan (AF2949; R&D Systems), syndecan-1 (AF3190; R&D Systems), PDGFR-α (AF1062; R&D Systems), PDGFR-β (AF1042; R&D Systems) and MMP9 (ab38898; Abcam) or human aggrecan (AF1220; R&D Systems), brevican (AF4009, R&D Systems), biglycan (AF2667; R&D Systems), decorin (AF143; R&D Systems), and glypican (AF4519; R&D Systems). After incubation for 1 h with specific anti-IgG secondary antibodies (Sigma-Aldrich, Vector Laboratories or R&D systems), conjugated to peroxidase, immunoreactive signals were visualized by enhanced chemiluminescence using ECL prime Western Blotting detection reagent (GE Healthcare) and a chemiluminescence documentation system (Uvitec). As glyceraldehyde 3-phosphate dehydrogenase (GAPDH) and actin are cleaved by HF3^11,54^ and hence could not be used as protein loading controls we show the electrophoretic profile of the skin proteins by SDS-PAGE (30 μg) run under the same conditions used for Western blot analysis and stained with colloidal Coomassie blue or Ponceau S (Supplementary Figure S4).

## Supplementary information

Supplementary Figures.

Supplementary Tables.

## Data Availability

The datasets generated during and/or analyzed during the current study are available from the corresponding author on reasonable request.

## Abbreviations

BM: Basement membrane
ECM: Extracellular matrix
MMP: Matrix metalloproteinase
MHD: Minimum hemorrhagic dose
PDGF: Platelet-derived growth factor
PDGFR: Platelet-derived growth factor receptor
SPR: Surface plasmon resonance
SVMP: Snake venom metalloproteinase
