# Supplementary material for: Cleavage of proteoglycans, plasma proteins and the platelet-derived growth factor receptor in the hemorrhagic process induced by snake venom metalloproteinases
Source: Sci Rep. 2020 Jul 31;10:12912. doi: 10.1038/s41598-020-69396-y (PMC7395112; doi:10.1038/s41598-020-69396-y)
Supplement: Supplementary file 1 — Supplementary Figures. [file 41598_2020_69396_MOESM1_ESM.pptx]

## Slide 1
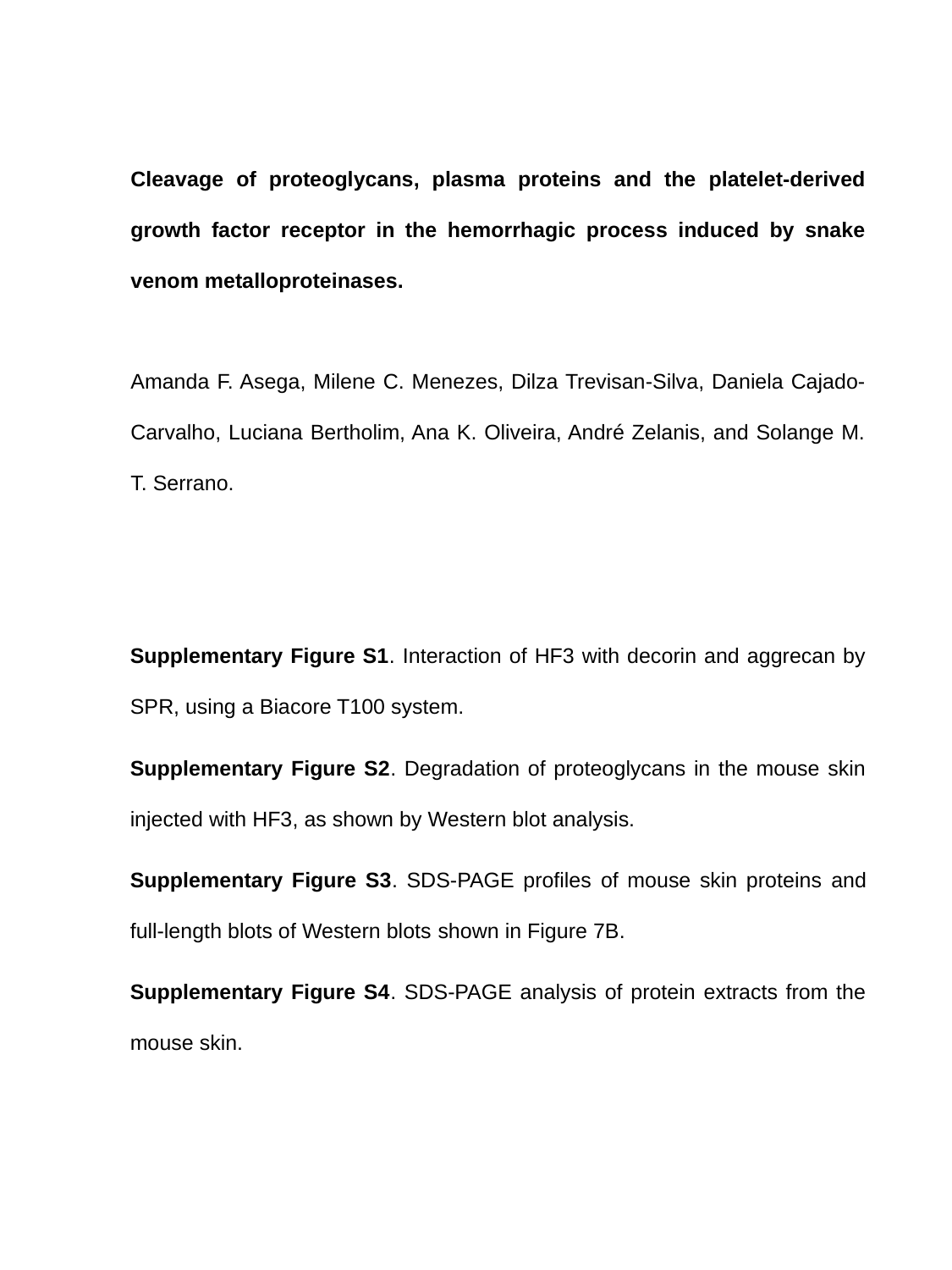

Cleavage of proteoglycans, plasma proteins and the platelet-derived growth factor receptor in the hemorrhagic process induced by snake venom metalloproteinases.
Amanda F. Asega, Milene C. Menezes, Dilza Trevisan-Silva, Daniela Cajado-Carvalho, Luciana Bertholim, Ana K. Oliveira, André Zelanis, and Solange M. T. Serrano.
Supplementary Figure S1. Interaction of HF3 with decorin and aggrecan by SPR, using a Biacore T100 system.
Supplementary Figure S2. Degradation of proteoglycans in the mouse skin injected with HF3, as shown by Western blot analysis.
Supplementary Figure S3. SDS-PAGE profiles of mouse skin proteins and full-length blots of Western blots shown in Figure 7B.
Supplementary Figure S4. SDS-PAGE analysis of protein extracts from the mouse skin.

## Slide 2
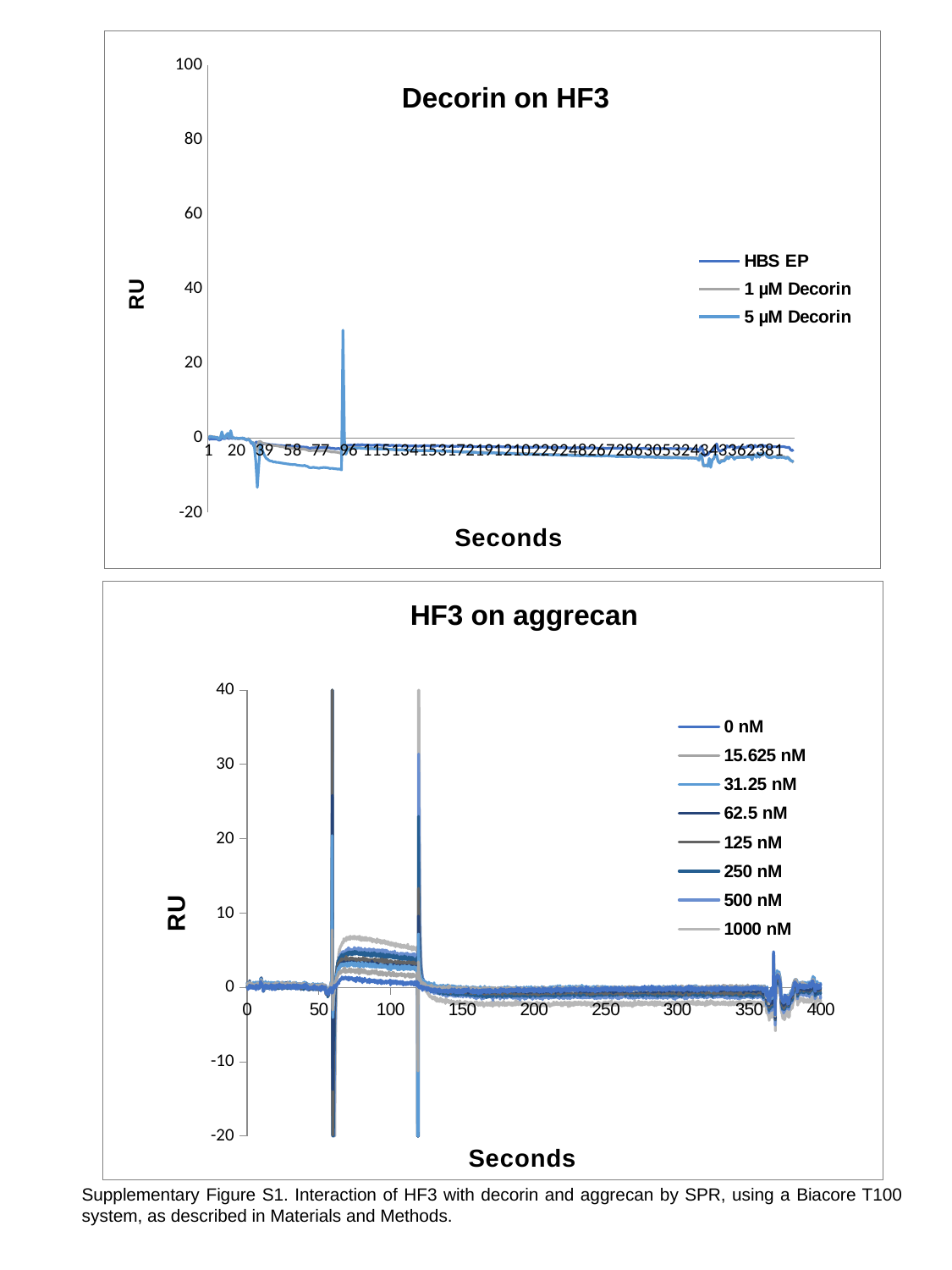

### Chart: Seconds
| Category | HBS EP | 1 µM Decorin | 5 µM Decorin |
|---|---|---|---|Decorin on HF3
HF3 on aggrecan
### Chart
| Category | 0 nM | Cycle=2_Fc=4-1_X | 15.625 nM | Cycle=3_Fc=4-1_X | 31.25 nM | Cycle=4_Fc=4-1_X | 62.5 nM | Cycle=5_Fc=4-1_X | 125 nM | Cycle=6_Fc=4-1_X | 250 nM | Cycle=7_Fc=4-1_X | 500 nM | Cycle=8_Fc=4-1_X | 1000 nM |
|---|---|---|---|---|---|---|---|---|---|---|---|---|---|---|---|Supplementary Figure S1. Interaction of HF3 with decorin and aggrecan by SPR, using a Biacore T100 system, as described in Materials and Methods.

## Slide 3
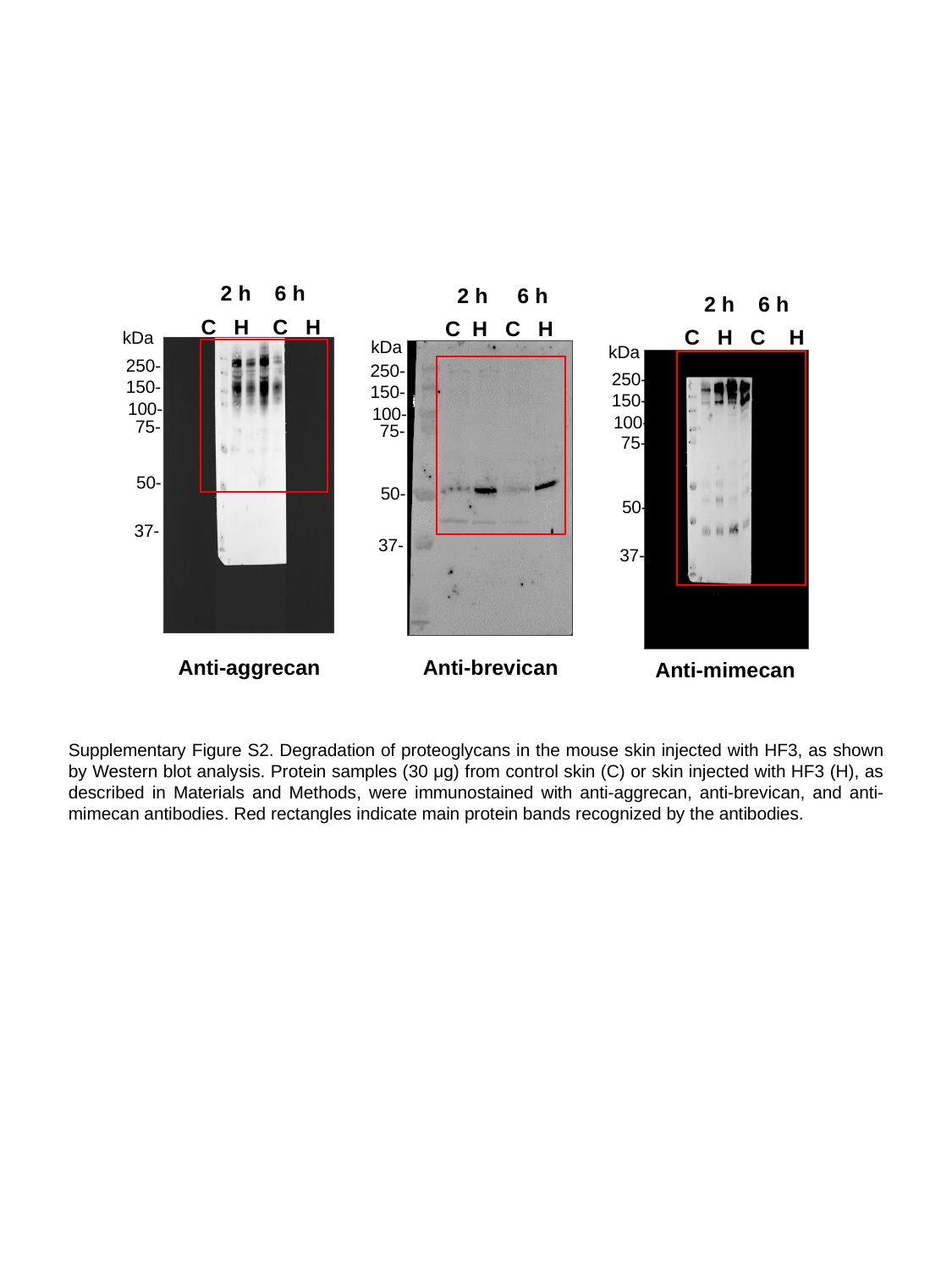

2 h 6 h
 C H C H
250-
150-
100-
75-
50-
37-
Anti-aggrecan
2 h 6 h
 C H C H
250-
150-
100-
75-
50-
37-
Anti-brevican
2 h 6 h
 C H C H
kDa
250-
150-
100-
75-
50-
37-
Anti-mimecan
kDa
kDa
Supplementary Figure S2. Degradation of proteoglycans in the mouse skin injected with HF3, as shown by Western blot analysis. Protein samples (30 μg) from control skin (C) or skin injected with HF3 (H), as described in Materials and Methods, were immunostained with anti-aggrecan, anti-brevican, and anti-mimecan antibodies. Red rectangles indicate main protein bands recognized by the antibodies.

## Slide 4
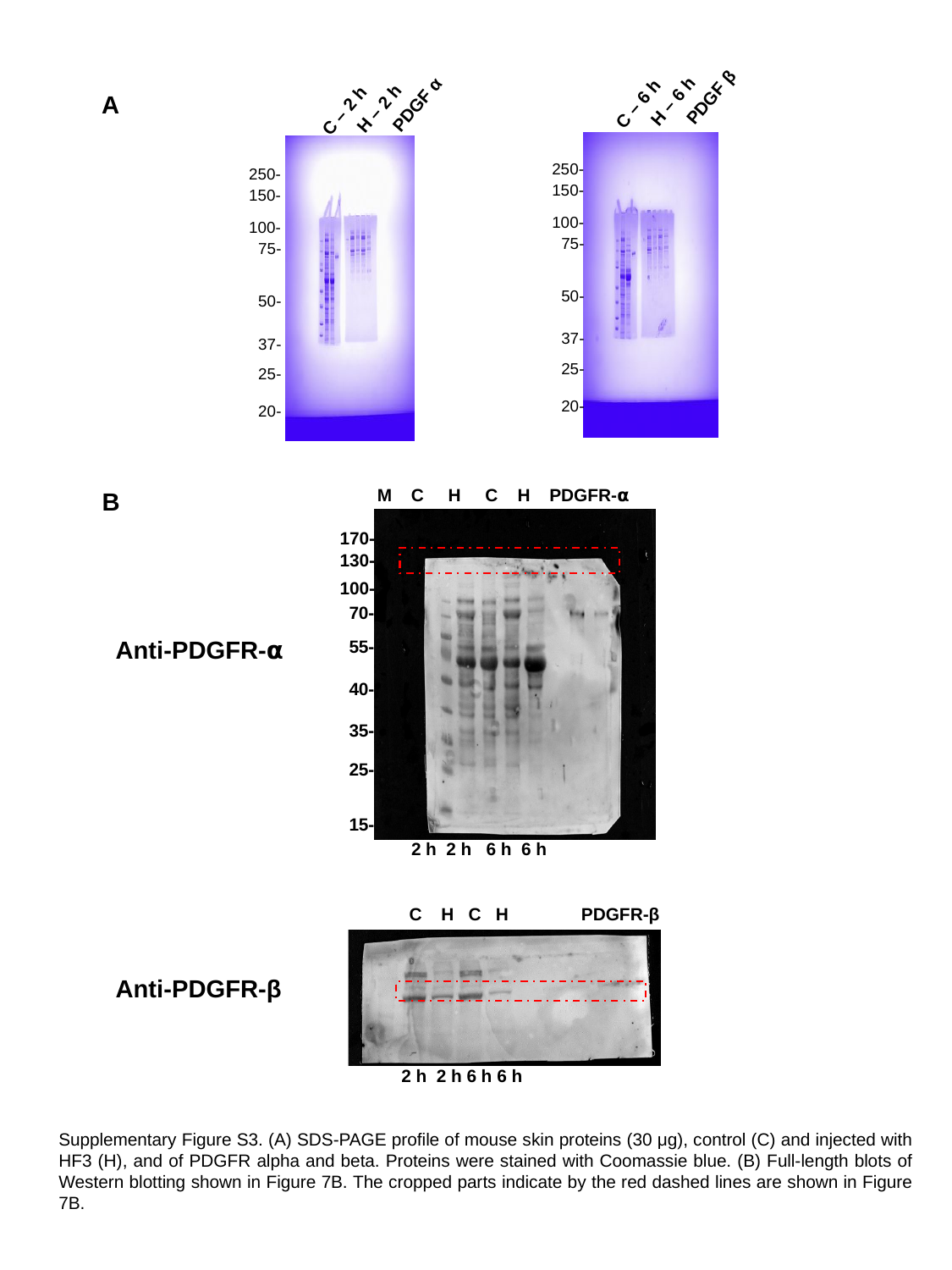

PDGF β
H – 6 h
C – 6 h
PDGF α
H – 2 h
C – 2 h
A
250-
250-
150-
150-
100-
100-
75-
75-
50-
50-
37-
37-
25-
25-
20-
20-
 M C H C H PDGFR-⍺
B
170-
130-
100-
70-
Anti-PDGFR-⍺
55-
40-
35-
25-
15-
 2 h 2 h 6 h 6 h
 C H C H PDGFR-β
Anti-PDGFR-β
 2 h 2 h 6 h 6 h
Supplementary Figure S3. (A) SDS-PAGE profile of mouse skin proteins (30 μg), control (C) and injected with HF3 (H), and of PDGFR alpha and beta. Proteins were stained with Coomassie blue. (B) Full-length blots of Western blotting shown in Figure 7B. The cropped parts indicate by the red dashed lines are shown in Figure 7B.

## Slide 5
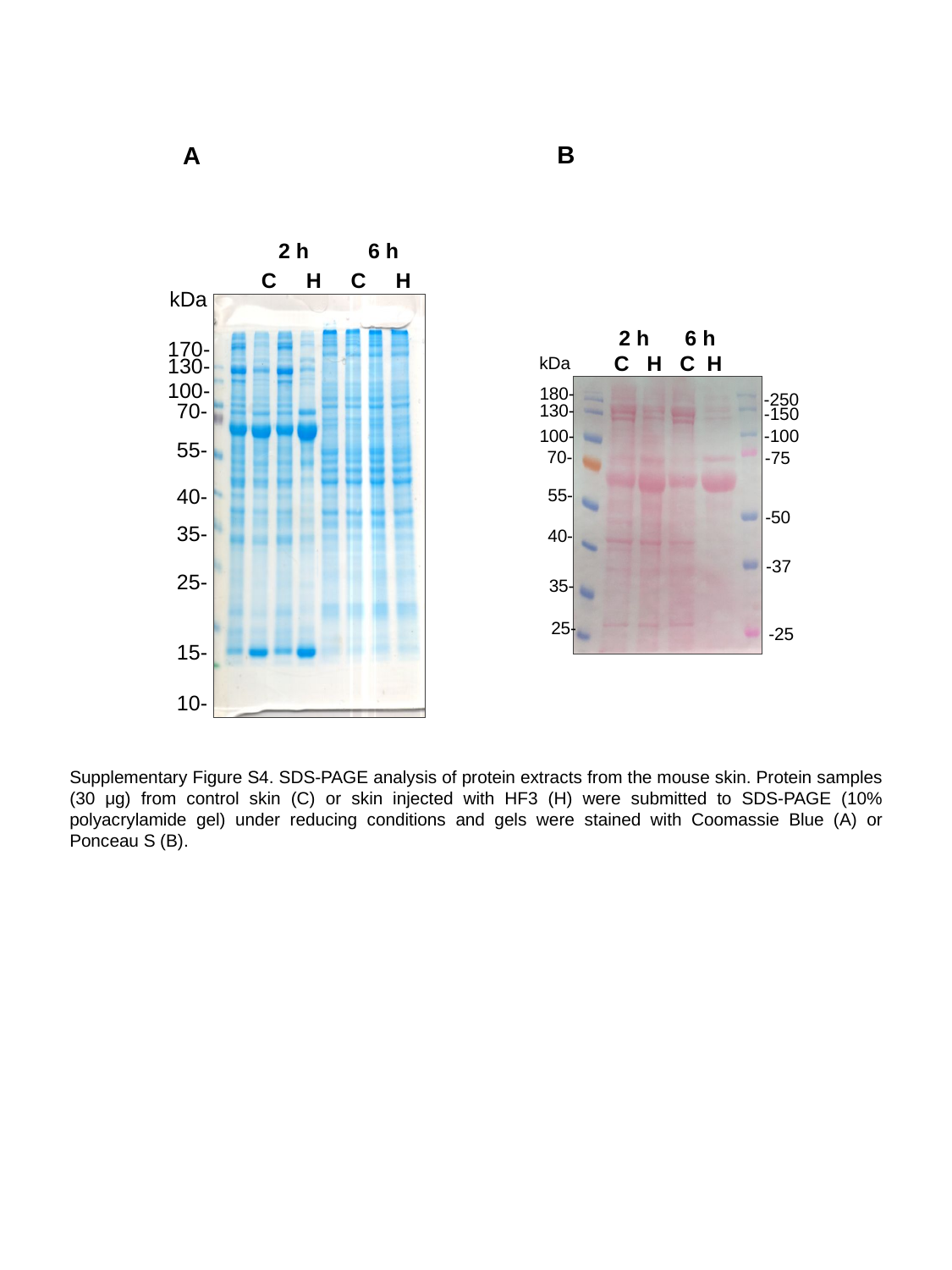

B
A
 2 h 6 h
 C H C H
kDa
2 h 6 h
170-
 C H C H
kDa
130-
100-
180-
-250
70-
130-
-150
100-
-100
55-
70-
-75
40-
55-
-50
35-
40-
-37
25-
35-
25-
-25
15-
10-
Supplementary Figure S4. SDS-PAGE analysis of protein extracts from the mouse skin. Protein samples (30 μg) from control skin (C) or skin injected with HF3 (H) were submitted to SDS-PAGE (10% polyacrylamide gel) under reducing conditions and gels were stained with Coomassie Blue (A) or Ponceau S (B).
